# Method development and Application of Growth Phase specific recombinant Protein Glycosylation Analysis

**DOI:** 10.1186/1753-6561-9-S9-P37

**Published:** 2015-12-14

**Authors:** Klaudia Grunwald, Thomas Noll, Heino Büntemeyer

**Affiliations:** 1 Institute of Cell Culture Technology, Bielefeld University, Germany

## Background

Heterogeneity in recombinant protein quality (e.g. glycosylation) can result from a) changes in cultivation conditions during a fermentation process, b) product degradation due to differing residence times of product molecules in the supernatant, c) heterogeneities in intracellular PTM (post transcriptional modification) machinery, or from a combination thereof. To minimize heterogeneity in product quality it is of importance to evaluate the impact of these different mechanisms. Therefore we developed and applied a protocol, which can be used for analysis of a spectrum of different recombinant proteins and production cell lines aiming to provide a tool that enables growth phase related product analytics, for monitoring variations of the product during different phases of a cultivation process. Important for growth phase specific product analysis is the possibility to separate recombinant proteins produced during different growth phases. Exchanging media without changing its composition regarding nutrient and waste products should result in growth characteristics comparable to a cultivation without media exchange.

## Materials and methods

An erythropoietin-producing CHO K1-derived cell line was cultivated in parallel with the host cell line in batch culture in chemically defined, animal component-free CHO growth media (Xell AG) for different cultivation periods. Furthermore the α-1-antitrypsin producing human AGE1.hn cell clone was cultivated simultaneously. At defined time points a media exchange was performed by replacing the supernatant of the production cell line, which contained the product, with supernatant of the host cell line. The supernatant of the production cell line was stored at - 20 °C for later analysis. Afterwards cultivation was continued until viability dropped below 50 %.Additionally, a control batch of the production cell line was performed without media exchange. All batches were carried out at least in two biological replicates. Cultivations with and without media exchange were compared in terms of to viable cell density and viability (CEDEX (Roche)), glucose and lactate levels (YSI (Xylem)) as well as amino acid concentrations (HPLC). Secreted recombinant erythropoietin from different fermentation phases was analyzed by capillary zone electrophorese (CZE, Kontron Instruments) to get an insight of the degree of sialylation, as crucial modification for product quality.

## Results

**Figure 1:**
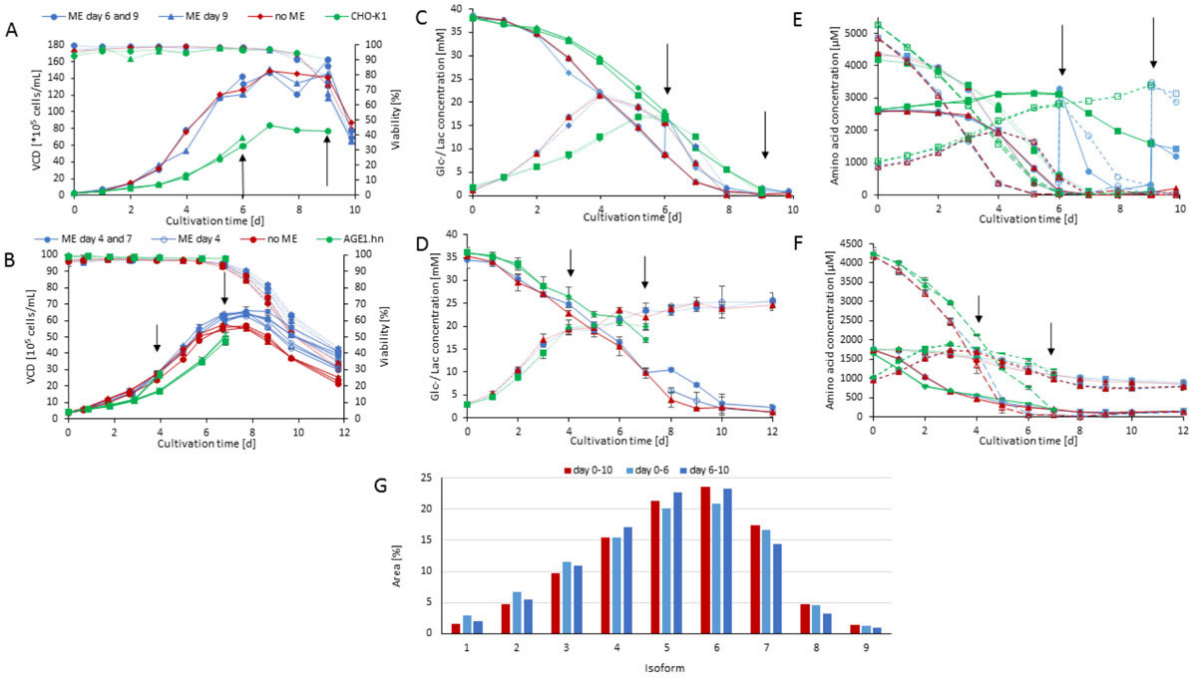
**Viable cell densities and viability of batch cultivations of (A) CHO-K1-EPO and CHO-K1 (B) AGE1**.hn-AAT and AGE1.hn; Glucose and lactate concentration of batch cultivations of (C) CHO-K1-EPO and CHO-K1 (D) AGE1.hn-AAT and AGE1.hn; Amino acid course of (E) CHO-K1-EPO and CHO-K1 (F) AGE1.hn-AAT and AGE1.hn batch cultivations: aspartate (-), asparagine (···), glutamine ( - -), glutamate(-- --); (G) Percentage of the nine most common isoforms of erythropoietin produced during different fermentation phases.

During the cultivation of CHO-K1-EPO and its host cell line, we could observe, that the production cell line reached higher cell densities (up to 156 × 105 cells/mL) than the host cell line (83 × 105 cells/mL) (Fig. 1 (A)). Therefore substrate concentrations like glucose and glutamine decreased earlier than during the cultivation of the host cell line (Fig. 1 (C), (E)).

On the contrary the cultivation of AGE1.hn and AGE1.hn-AAT ran much more similar (Fig. 1 (B)). Glucose, lactate (Fig. 1 (D)) and the amino acid data (Fig. 1 (F)) show similar trends comparing the host cell line with the production cell line the production cell line with and without media exchange.

In Fig. 1 (B) the results of a CZE of recombinant erythropoietin produced in different phases are shown. We detected nine isoforms due to different degrees of sialylation of the N- and O-glycans, as described for recombinant erythropoietin before [1]. In a later fermentation phase little effects on sialylation (isoforms seven, eight and nine)are obvious. At day nine of the cultivation the viability decreased from 90 % to under 80 %, and on day ten the viability was below 50 %. Sialylases accumulating in the supernatant due to cell lysis, may be responsible for the decline of product quality by reducing the amount of sialic acids [2,3].

## Conclusions

Fermentation phase specific sampling with media exchanges at similar cell densities shows no changes neither in growth behavior nor glucose, lactate and amino acid metabolism. For this procedure it is important, that the nutrient content of the conditioned media is similar to the media to be exchanged. For a production cell line which growth behavior differs highly from the host cell line the cultivation needs to be adapted, or supernatants from different fermentation phases have to be prepared in advance for proper utilization at time of medium exchange.

Differential analysis of product heterogeneity relating to different fermentation phases is possible without any labeling of substrates, Glycosylation (N-glycans, O-glycans), degree of sialylation or the ratio of isoforms, all of high importance for product quality, can be analyzed easily in this way.

In this case, most of the product heterogeneity of erythropoietin produced by CHO-K1-EPO seems to be based on the cell itself. Apparently only few variations are due to different residence times.

In addition to this fermentation phase dependent sampling, the impact of different residence times on products in media as well as in supernatants needs to be analyzed to gain a deeper insight into reasons for product heterogeneity. With these handy experiments factors for variable product heterogeneity can be identified for every product and cell line to optimize the cell, the media and the process.

## References

[B1] European Pharmakopoeia Europarat 8.0 2014

[B2] Berger M Protein Glycosylation and Its Impact on Biotechnology Adv Bio chem Eng Biotechnol 2012 127 165 185 10.1007/10_2011_101 21975953

[B3] Gramer M Product Quality Considerations for Mammalian Cell CultureProcess Development and Manufactoring AdvBiochemEngBiotechnol 2014 139 123 166 10.1007/10_2013_214 23748351

